# Diagnostic yield of colon capsule endoscopy for Crohn’s disease lesions in the whole gastrointestinal tract

**DOI:** 10.1186/s12876-021-01657-0

**Published:** 2021-02-16

**Authors:** Keisaku Yamada, Masanao Nakamura, Takeshi Yamamura, Keiko Maeda, Tsunaki Sawada, Yasuyuki Mizutani, Eri Ishikawa, Takuya Ishikawa, Naomi Kakushima, Kazuhiro Furukawa, Eizaburo Ohno, Hiroki Kawashima, Takashi Honda, Masatoshi Ishigami, Mitsuhiro Fujishiro

**Affiliations:** 1 Department of Gastroenterology and Hepatology, Nagoya University Graduate School of Medicine, 65 Tsurumai-cho, Syowa-ku, Nagoya City, Aichi Japan; 2 Department of Endoscopy, Nagoya University Hospital, 65 Tsurumai-cho, Showa-ku, Nagoya, 466-8550 Japan

**Keywords:** Colon capsule endoscopy, Crohn’s disease, Diagnostic yield, Retention, Whole gastrointestinal tract, Prospective study

## Abstract

**Background:**

Crohn’s disease (CD) can involve the upper gastrointestinal (GI) tract as well as the small and large bowel. PillCam colon capsule endoscopy (PCCE-2) enables observation of the whole GI tract, but its diagnostic yield for CD lesions in the whole GI tract remains unknown.

**Aim:**

To elucidate the diagnostic yield of PCCE-2 in patients with CD.

**Methods:**

Patients with CD who underwent PCCE-2 and double-balloon endoscopy (DBE) using oral and anal approaches were evaluated for CD lesions in the whole GI tract. We divided the small bowel into three segments (jejunum, ileum, and terminal ileum), and the large bowel into four segments (right colon, transverse colon, left colon, rectum). Detection of ulcer scars, erosion, ulcers, bamboo joint-like appearance, and notch-like appearance was assessed in each segment. The diagnostic yield of PCCE-2 was analyzed based on the DBE results as the gold standard.

**Results:**

Of the total 124 segments, the sensitivities of PCCE-2 for ulcer scars, erosion, and ulcers were 83.3%, 93.8%, and 88.5%, respectively, and the specificities were 76.0%, 78.3%, and 81.6%, respectively. For the 60 small bowel segments, the sensitivities were 84.2%, 95.5%, and 90.0%, respectively, and the specificities were 63.4%, 86.8%, and 87.5%, respectively. For the 64 large bowel segments, the sensitivities were 80.0%, 90.0%, and 83.3%, respectively, and the specificities were 84.7%, 72.2%, and 77.6%, respectively.

**Conclusion:**

PCCE-2 provides a high diagnostic yield for lesions in the whole GI tract of patients with CD. Thus, we recommend its use as a pan-enteric tool in clinical settings.

## Introduction

Crohn’s disease (CD) is a chronic inflammatory bowel disease (IBD) that mainly involves the small and large bowel. The goals of treatment in CD have evolved in recent years from symptom control to healing of mucosal lesions visualized on endoscopy [1]. Mucosal healing has been associated with improved clinical outcomes, including sustained steroid-free clinical remission, decreased rates of surgery and hospitalization, reduced occurrence of new perianal complications, as well as improvement in quality of life and increased work productivity [2, 3]. With the development of small bowel capsule endoscopy (SBCE), direct endoscopic examination of the whole small bowel mucosa is available with high diagnostic yield [4–6]. However, in addition to SBCE, colonoscopy (CS) is required to evaluate the large-bowel mucosa, particularly in the case of ileocolonic CD. The introduction of a minimally invasive and high-performing tool for evaluating the whole gastrointestinal (GI) tract is highly anticipated, because even with balloon-assisted enteroscopy, it can be challenging to examine the whole GI tract.

The second-generation PillCam colon capsule endoscope (PCCE-2; Medtronic Co. Ltd., Dublin, Ireland) has been developed and can be used to examine the whole GI tract, including the small and large bowel, although it was originally designed as a tool to diagnose colorectal lesions [6]. PCCE-2 is a noninvasive procedure that enables visualization of the GI tract without sedation or gas insufflation [7]. The feasibility and safety of its use in colonic assessment have been investigated for polyps and cancer [8–11]. Furthermore, PCCE-2 has been clinically applied as a tool to replace CS for observing the large bowel in patients with ulcerative colitis (UC) [10], and an original preparation regimen has been developed [11, 12]. Although some studies confirmed the safety and feasibility of PCCE-2 as a pan-enteric tool for patients with CD [6, 13], the diagnostic yield of PCCE-2 for the whole GI CD lesions remains unknown. On the other hand, balloon-assisted enteroscopy, such as double-balloon endoscopy (DBE) and single-balloon endoscopy (SBE), has been shown to have a high diagnostic yield in detecting small bowel diseases [14]. SBE results have been reported as the gold standard for small bowel lesions of CD [15]. Therefore, the aim of this study was to prospectively elucidate the diagnostic yield of PCCE-2 for the whole GI CD lesions in reference to DBE results.

## Materials and methods

### Patients

From June 2018 to August 2019, patients who were scheduled for DBE for assessment of CD activity at the Nagoya University Hospital were enrolled in this prospective study. The study protocol was approved by the local ethics committee (Nagoya University IRB 2015–372) and registered at UMIN-CTR (UMIN000019632). Patients provided informed consent. This study was conducted in accordance with the principles of the Declaration of Helsinki.

### Study protocol

The details of the study protocol are shown in Table 1. On the first day, transoral DBE was performed, and patients who showed no stricture on DBE proceeded to PCCE-2 preparation. In addition to PCCE-2 preparation, patency of the GI tract was assessed using the PillCam patency capsule (PC) (Medtronic Co. Ltd., Dublin, Ireland). PCCE-2 was not performed for patients in whom patency was not confirmed. After the excretion of PCCE-2, trans-anal DBE was performed. The primary endpoint of this study was the diagnostic yield of PCCE-2 for erosive lesions in each segment, and the secondary endpoints were the evaluation of CD activity using PCCE-2 and clinical results of PCCE-2 including retention of capsule.

**Table 1:** Original regimen of PCCE-2 plus DBE examinations

Day	Time	Procedure
Day 1	Daytime	DBE oral approach
	Before bedtime	2 senna tablets and PC
Day 2	Before bedtime	Magnesium citrate 50 g (180 ml): hypertonic method 2 senna tablets
Day 3	8:30	1.0 L PEG and patency confirmation
	10:00	Capsule ingestion with mosapride citrate 20 mg
	1st boost (when the PCCE-2 reaches the small intestine)	A mixture of PEG 1000 ml and water 1000 ml Castor oil 30 ml
	2nd boost	Sodium picosulfate 48 mg Castor oil 30 ml
	3rd boost	Magnesium citrate 50 g (900 ml): isotonic method
	(after the excretion of PCCE-2)	DBE anal approach

*PCCE-2* the second-generation PillCam colon capsule endoscopy, *DBE* double-balloon endoscopy, *PC* patency capsule, *PEG* polyethylene glycol

### Definitions of DBE and PCCE-2 recordings

**Fig. 1:**
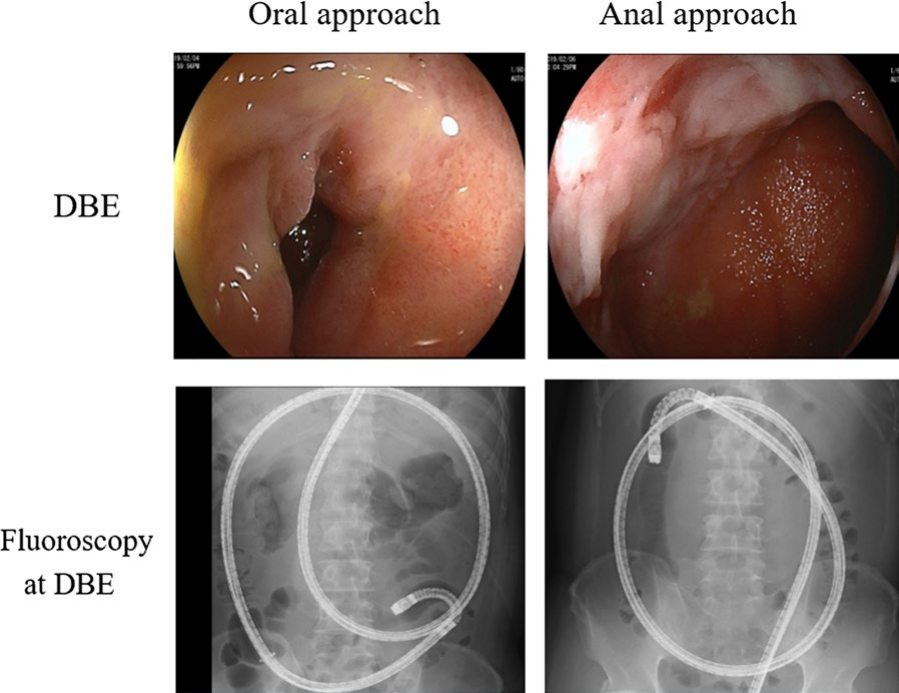
The landmark Crohn’s disease lesion is observed by double-balloon endoscopy (DBE) with oral and anal approaches (top). Whole gastrointestinal tract observation is achieved using fluoroscopy (bottom)

The whole GI tract was observed on both DBE and PCCE-2 recordings. Whole GI tract observation with DBE was defined as when the landmark CD lesion was observed using both oral and anal approaches, or several endoscopists judged that DBE enabled visualization of the whole GI tract using fluoroscopy and additional gastrografin enterography during DBE, according to previous studies [16, 17] (Fig. 1). In cases where the CCE was not excreted, CD lesions were analyzed as far as the CCE reached. Two readers who were blinded to the clinical background and DBE findings of the patient analyzed each PCCE-2 video.

**Fig. 2:**
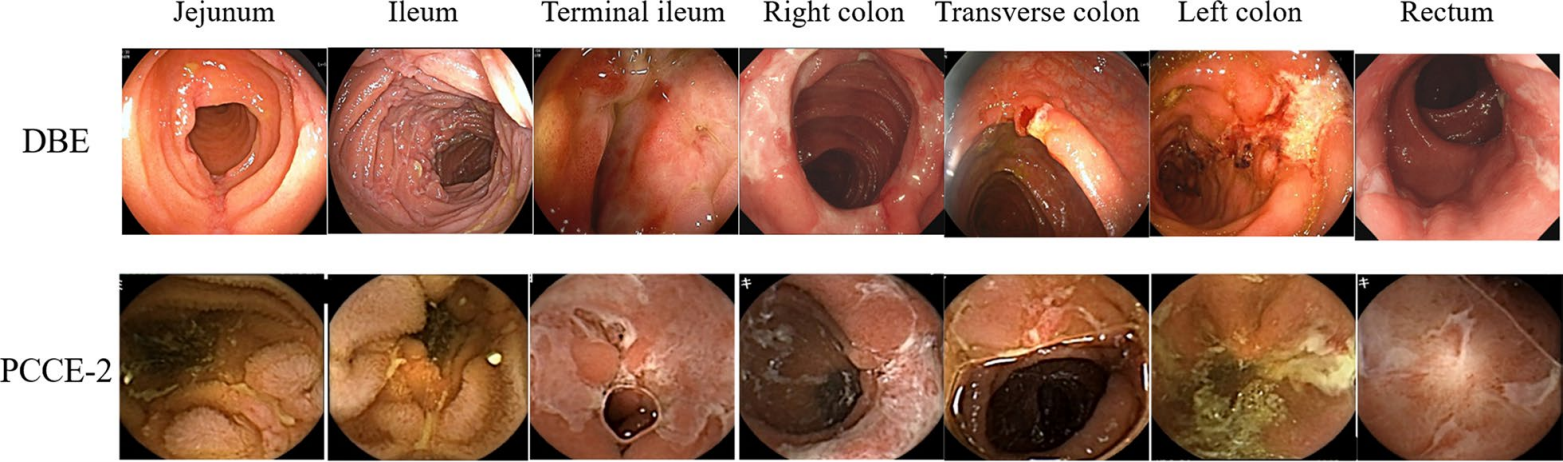
Images of double-balloon endoscopy (top) and the corresponding second-generation PillCam colon capsule endoscopy (PCCE-2) image (bottom) of the ulcer lesion

We divided the small bowel into three segments, namely, the jejunum, ileum, and terminal ileum, and the large bowel into four segments, namely, the right side of the colon (cecum, ascending colon), transverse colon, and left side of the colon (descending colon, sigmoid colon), and rectum (Fig. 2). The terminal ileum was defined as the Sect. 10 cm from the ileocecal valve on DBE, and the video segment 5 min before the cecum was reached on PCCE-2. In the small bowel postoperative cases, the remaining bowel was divided into three segments as previously defined. The diagnostic yield of PCCE-2 for the presence of ulcer scars, erosion, and ulcers in each segment was evaluated in the seven segments with the DBE results defined as the reference gold standard. We focused on evaluating the presence of any lesion in each segment. In cases with several lesions in one segment, we confirmed that the segment was positive. The number of positive segments in DBE and PCCE-2 were compared. PCCE-2 findings were also evaluated for esophageal and gastric lesions using the same strategy. According to CD activity, SES-CD was evaluated right after DBE. SES-CD originally involves the terminal ileum and rectum, and in this study, it was applied for the jejunum and ileum using the same evaluation method. Total score, the sum of SES-CD in each segment, was described as modified SES-CD. Capsule endoscopy Crohn’s disease activity index (CECDAI), frequently used to evaluate CD activity in small bowel capsule endoscopy, was used to evaluate the seven segments. The total score, the sum of CECDAI in each segment of PCCE-2, was described as modified CECDAI in this study. The relation between modified SES-CD and modified CECDAI was analyzed in the same patients.

### Colon cleansing level

Colon cleanliness was determined in accordance with a four-point grading scale (excellent, good (categorized as adequate), fair, and poor (categorized as inadequate)) as reported in a previous study [18].

### Statistical analysis

All data were analyzed using SPSS version 24.0 statistical software (IBM, Tokyo, Japan). Differences in each segment of the small and large bowel were analyzed using Fisher’s exact test. The factors that influenced the incomplete PCCE-2 were analyzed using a logistic regression model. To analyze the relation between modified SES-CD and modified CECDAI, Spearman’s rank correlation coefficient was used. Differences with a *p*-value < 0.05 were considered statistically significant.

## Results

### Patients

A total of 22 patients were enrolled and underwent DBE using an oral approach. Small bowel obstruction was suspected in one patient, and patency was not confirmed by PC in another patient. Finally, 20 patients underwent PCCE-2 and subsequent DBE using an anal approach. Table 2 shows the characteristics of the 20 patients.

**Table 2:** Patient characteristics (N = 20)

Age (years)	Median, range	35 (19–67)
Sex	Male/female	15/5
Disease duration (years)	Median, range	10 (1–22)
Disease location	L1/L2/L3^a^	6/0/14
CDAI	Median, range	142 (60–324)^b^
History of surgery (%)		70.0% (14/20)
Medication (%)	Mesalazine	80.0% (16/20)
	Elemental diet	70.0% (14/20)
	Anti-TNF agents	55.0% (11/20)
	Thiopurines	25.0% (5/20)
	Ustekinumab	10.0% (2/20)

*CDAI* Crohn’s disease activity index, *TNF* tumor necrosis factor

^a^L1/L2/L3: ileal/colonic/ileocolonic, Montreal classification

^b^CDAI could not be evaluated in one patient because of the presence of stoma

### Results of the PCCE-2 procedure and number of segments evaluated by both modalities

The PCCE-2 excretion rate within the battery life was 75% (15/20). Of the five patients who did not excrete the PCCE-2, two were observed up to the left colon, one was observed up to the transverse colon, and two were observed up to the right colon. Of the 15 patients who excreted the PCCE-2, the median duration of the entire examination was 455 min, the gastric transit time was 80 min, the small intestinal transit time was 69 min, and the colorectal transit time was 265 min. The colon cleansing level was evaluated as adequate in 80% of patients.

Of the 20 patients, 20 gastric, 60 small bowel, and 64 large bowel segments were evaluated. As for large bowel segments, 16 segments were excluded because PCCE-2 could not be observed or evaluated in postoperative cases.

### Positive findings of PCCE-2 in the whole GI tract

Various lesions, including ulcer scars, erosion, ulcers, bamboo joint-like appearance, and notch-like appearance, were detected in the whole GI tract of patients with CD by PCCE-2. Among them, ulcer scars, erosion, and ulcers were frequently observed in the small and large bowel, and erosion and bamboo joint-like appearance were most commonly observed in the stomach. The detection rates of PCCE-2 for ulcer scars, erosion, and ulcers per segment were 52%, 43.3%, and 10% in the small bowel, and 20%, 38%, and 28%, respectively, in the large bowel. The detection rates for erosion and bamboo joint-like appearance in the stomach were 35% and 10%, respectively.

### Diagnostic yield of PCCE-2

The diagnostic yield of PCCE-2 for the small and large bowel is shown in Table 3. The PCCE-2 sensitivities for ulcer scars, erosion, and ulcers were 83.3%, 93.8%, and 88.5%, respectively, and the specificities were 76.0%, 78.3%, and 81.6%, respectively.

**Table 3:** Diagnostic yield of PCCE-2 for the small and large bowel

	Ulcer scar	Erosion	Ulcer
Sensitivity	83.3% (20/24)	93.8% (30/32)	88.5% (23/26)
Specificity	76.0% (76/100)	78.3% (72/92)	81.6% (80/98)
PPV	45.5% (20/44)	60.0% (30/50)	56.1% (23/41)
NPV	95.0% (76/80)	97.3% (72/74)	96.4% (80/83)
Accuracy	77.4% (96/124)	82.3% (102/124)	83.1% (103/124)

*PPV* positive predictive value, *NPV* negative predictive value, *PCCE-2* the second-generation PillCam colon capsule endoscopy

The diagnostic yield of PCCE-2 for the small bowel is shown in Table 4. Sensitivities and specificities for active CD lesions, such as erosion and ulcers, were more than 85%. No significant difference was found in the sensitivities and specificities between the three segments of the small bowel (Table 5).

**Table 4:** Diagnostic yield of PCCE-2 for the small bowel

	Ulcer scar	Erosion	Ulcer
Sensitivity	84.2% (16/19)	95.5% (21/22)	90.0% (18/20)
Specificity	63.4% (26/41)	86.8% (33/38)	87.5% (35/40)
PPV	51.6% (16/31)	80.8% (21/26)	78.3% (18/23)
NPV	89.7% (26/29)	97.1% (33/34)	94.6% (35/37)
Accuracy	70.0% (42/60)	90.0% (54/60)	88.3% (53/60)

*PPV* positive predictive value, *NPV* negative predictive value, *PCCE-2* the second-generation PillCam colon capsule endoscopy

**Table 5:** Diagnostic yield of PCCE-2 for the small bowel by segment

	Jejunum	Ileum	Terminal ileum	*p* value
*Ulcer scar*				
Sensitivity	100% (5/5)	75.0% (6/8)	83.3% (5/6)	n.s.*
Specificity	53.3% (8/15)	66.7% (8/12)	71.4% (10/14)	n.s.*
*Erosion*				
Sensitivity	100% (4/4)	90.0% (9/10)	100% (8/8)	n.s.*
Specificity	93.8% (15/16)	90.0% (9/10)	75.0% (9/12)	n.s.*
*Ulcer*				
Sensitivity	100% (4/4)	87.5% (7/8)	87.5% (7/8)	n.s.*
Specificity	87.5% (14/16)	83.3% (10/12)	83.3% (10/12)	n.s.*

*PCCE-2* the second-generation PillCam colon capsule endoscopy

*Fisher’s exact test

**Fig. 3:**
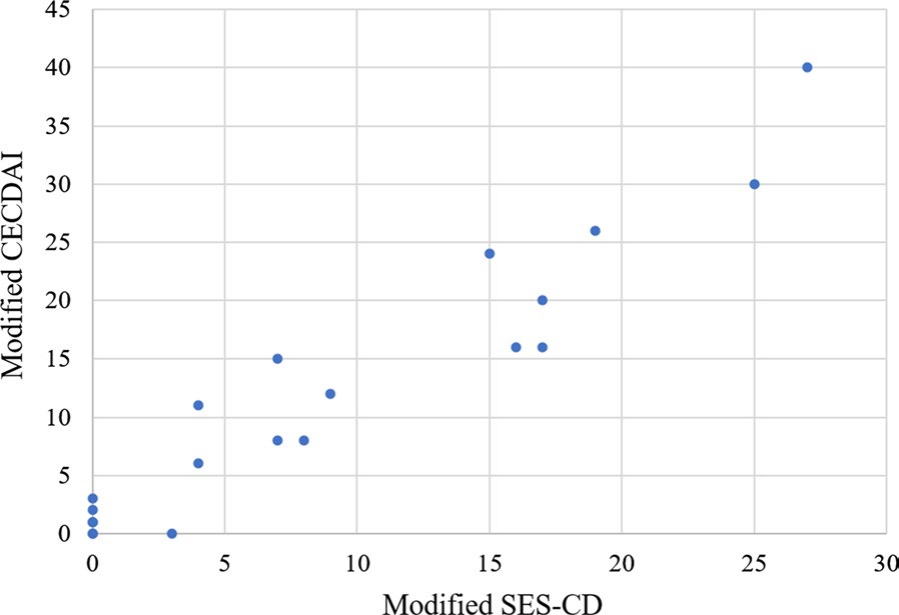
The relation between modified SES-CD and modified CECDAI. Modified CECDAI was well related to modified SES-CD (Spearman’s rank correlation coefficient, rho = 0.935, *p* < 0.001)

Table 6 shows the diagnostic yield of PCCE-2 for the large bowel. The sensitivities were satisfactory, but the specificities for erosion and ulcers were relatively low compared with those of the small bowel. No significant difference was observed between the four segments of the large bowel, as was found for the small bowel (Table 7). Modified CECDAI was well related to modified SES-CD (Fig. 3) (Spearman’s rank correlation coefficient, rho = 0.935, *p* < 0.001).

**Table 6:** Diagnostic yield of PCCE-2 for the large bowel

	Ulcer scar	Erosion	Ulcer
Sensitivity	80.0% (4/5)	90.0% (9/10)	83.3% (5/6)
Specificity	84.7% (50/59)	72.2% (39/54)	77.6% (45/58)
PPV	30.8% (4/13)	37.5% (9/24)	27.8% (5/18)
NPV	98.0% (50/51)	97.5% (39/40)	97.8% (45/46)
Accuracy	84.4% (54/64)	75.0% (48/64)	78.1% (50/64)

*PPV* positive predictive value, *NPV* negative predictive value, *PCCE-2* the second-generation PillCam colon capsule endoscopy

**Table 7:** Diagnostic yield of PCCE-2 for the large bowel by segment

	Right colon	Transverse colon	Left colon	Rectum	*p* value
*Ulcer scar*					
Sensitivity	100% (1/1)	50% (1/2)	100% (1/1)	100% (1/1)	n.s.*
Specificity	86.7% (13/15)	86.7% (13/15)	86.7% (13/15)	84.6% (11/13)	n.s.*
*Erosion*					
Sensitivity	100% (3/3)	100% (3/3)	66.7% (2/3)	100% (1/1)	n.s.*
Specificity	69.2% (9/13)	64.3% (9/14)	78.6% (11/14)	76.9% (10/13)	n.s.*
*Ulcer*					
Sensitivity	100% (1/1)	66.7% (2/3)	100% (1/1)	100% (1/1)	n.s.*
Specificity	80.0% (12/15)	78.6% (11/14)	75.0% (12/16)	76.9% (10/13)	n.s.*

*PCCE-2* the second-generation PillCam colon capsule endoscopy

*Fisher’s exact test

For gastric lesions, erosion and bamboo joint-like appearance were detected. Additional file 1: Table S1 shows the diagnostic yield of these gastric lesions. No esophageal lesions were noted in any of the patients.

### Incidence and severity of PCCE-2 procedure-related adverse events

PCCE-2 retention was not observed in patients with CD in whom patency was confirmed by PC. Of the 20 patients, one patient had moderate and three had mild abdominal bloating, two had mild abdominal pain, and two had mild nausea during PCCE-2 examination. Eighteen of the 20 patients indicated that they would undergo PCCE-2 again, and they preferred PCCE-2 to CS and DBE.

## Discussion

PCCE-2 can observe CD lesions that involve the whole GI tract in a single examination. Although some studies have reported the safety and feasibility of PCCE-2 for patients with CD [13, 15], the diagnostic yield of PCCE-2 for CD lesions of the whole GI is still unknown. This study is the first to elucidate the diagnostic yield of PCCE-2 for the whole GI tract. We demonstrated that PCCE-2 has high diagnostic yield for CD lesions of the whole GI.

With regard to the small bowel, a number of studies have already reported on the diagnostic yield of SBCE for small bowel CD lesions. The results of these studies vary with sensitivities of approximately 80% and specificities of approximately 50–75% [19, 20]. Solem et al*.* [19] reported that the specificity for small bowel CD lesions is significantly lower with SBCE than with other small bowel imaging modalities, such as CT enterography, ileocolonoscopy, and small bowel follow-through. We showed that the sensitivity and specificity of PCCE-2 for small bowel ulcer lesions were 90.0% and 87.5%, respectively. We can conclude that the specificity of PCCE-2 is high, suggesting that PCCE-2 may reduce false positives. The higher diagnostic accuracy of PCCE-2 has several potential explanations. First, the PCCE-2 has two head cameras, each with a 172° angle of view, allowing for almost 360° visual coverage of the colon. Second, PCCE-2 has improved image acquisition and adaptive frame rates of 4 to 35 images per second [21, 22], which are much higher than the 2 to 6 image frame rates of SBCE. Therefore, the performance of PCCE-2 could improve the diagnostic yield for small bowel lesions. Furthermore, the use of laxatives has been reported to be beneficial in patients likely to have subtle findings on SBCE because laxatives improve small bowel visualization quality [23]. In this study, the regular bowel preparation before PCCE-2 ingestion may also have contributed to the improved diagnostic yield.

**Fig. 4:**
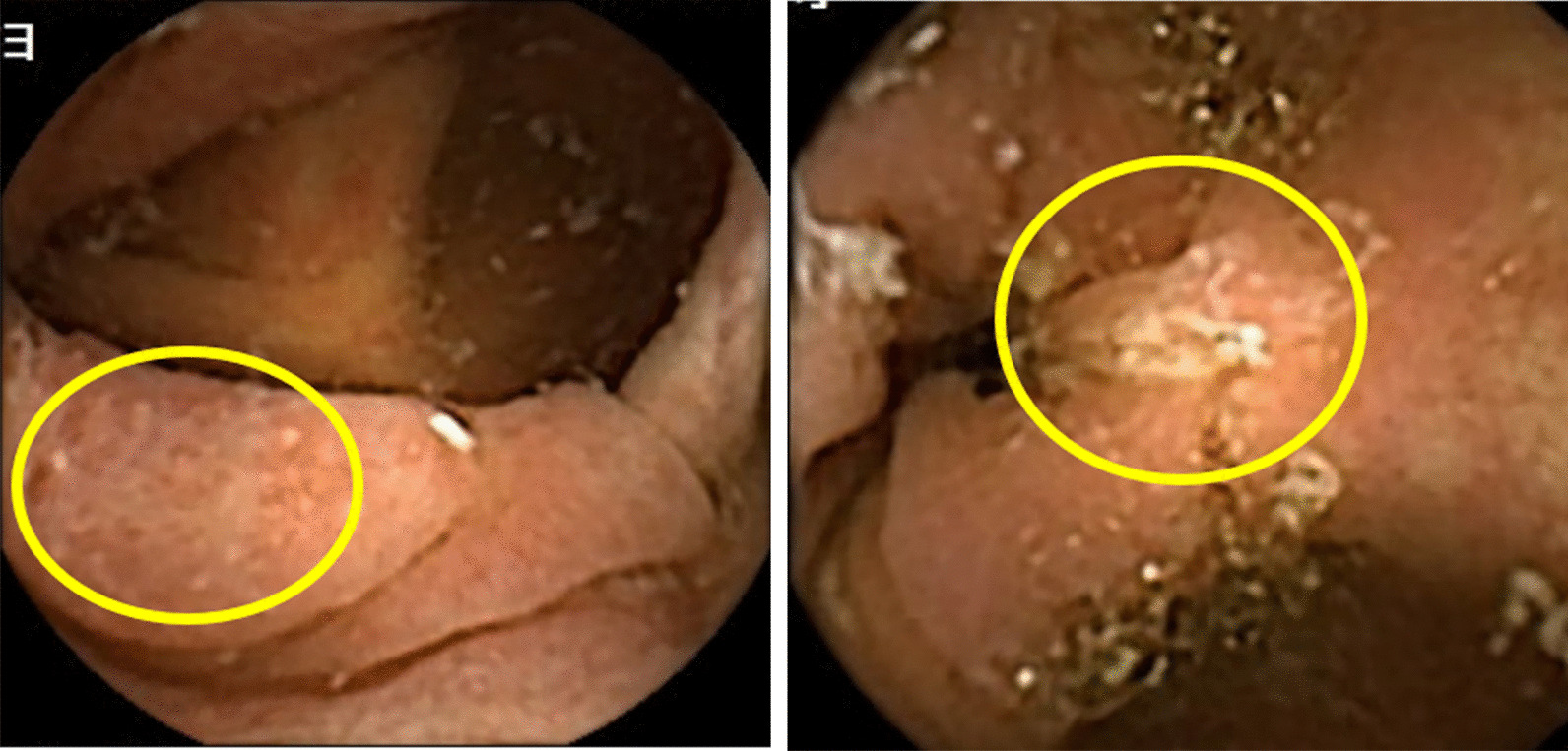
False-positive cases: stools may be erroneously identified as erosion and ulcer

The specificities of PCCE-2 for erosion and ulcers of the large bowel were 72.2% and 77.6%, respectively, which were lower than those of the small bowel. D'Haens et al*.* [1] stated that the low specificity may be related to bowel preparation, with adherent stools being erroneously identified as ulcerations (Fig. 4). With regard to the diagnostic yield of the large bowel, there is still room for improvement.

In addition to the observation of the small and large bowel, PCCE-2 can also observe upper GI lesions simultaneously. In patients with CD, gastric lesions, such as erosion, ulcers, and bamboo joint-like appearance, are detected at a relatively high frequency (24–73%) [24], and bamboo joint-like appearance is a characteristic finding in the upper GI. Fujiya et al*.* [25] reported that the detection rates of this finding are 38.3% in patients with CD, 2.5% in gender- and age-matched patients without IBD, and 1.5% in patients with UC. Therefore, PCCE-2 may be helpful in diagnosing patients with CD by distinguishing them from patients without IBD and patients with UC.

Furthermore, we demonstrated that the PCCE-2 procedure is safe for patients with CD. CD is a chronic inflammatory disease, and patients with CD need to undergo repeated GI examinations. Therefore, examination tools that are highly acceptable with fewer complications are desired. PCCE-2 retention was not observed in patients with CD in whom patency was confirmed by PC, and a high percentage of patients (18/20) indicated that they would undergo PCCE-2 again and preferred PCCE-2 to CS and DBE.

The use of castor oil (Himashi Oil; Yoshida Pharmaceutical, Tokyo, Japan) as part of the PCCE-2 regimen has been widely established as the standard regimen for bowel preparation because it improves capsule excretion rate and shortens capsule transit time [26, 27]. Although castor oil was also used in this study, the excretion rate of PCCE-2 was not sufficient. This was attributed to the limited PCCE-2 examination time because DBE using an anal approach had to be performed later. Another reason was inflammation of the GI tract due to CD. In patients with UC, the presence of colonic mucosal inflammation has been reported to correlate with longer PCCE-2 transit times because inflammation decreases the motility of the PCCE-2 [12]. Additional file 1: Table S2 shows the modified SES-CD score for small and large bowel inflammation (details are described in Additional file 1: Table S2), which was identified as a factor resulting in incomplete PCCE-2. Therefore, PCCE-2 seemed to be less likely to be excreted in patients with CD with an active lesion compared with patients with UC. Furthermore, postoperative cases tended to be classified more often to the non-excretion group, despite the short bowel. A possible reason was that the PCCE-2 moved around in a few cases for several hours at the anastomosis and did not flow to the anal side because of local intestinal peristalsis.

However, in contrast to UC that affects the colon in a retrograde and continuous manner starting from the rectum and extending proximally [28], CD involves discontinuous lesions, and the most common site is the terminal ileum [29]. Although five patients with CD did not excrete the PCCE-2 in this study, the terminal ileum could be observed in all cases, and no CD lesions were observed on DBE at the segment that could not be observed by PCCE-2. Therefore, even if the PCCE-2 is not excreted, the mucosal evaluation of CD is considered sufficient as PCCE-2 contributes to the evaluation of CD activity in cases with available PCCE-2.

### Limitations

The main limitation of this study was the small number of patients enrolled and the low number of patients with large bowel CD lesions. However, no studies have compared PCCE-2 results with DBE findings for the whole GI tract in patients with CD. Therefore, this study contributes significantly to the evidence supporting the clinical usefulness of the PCCE-2 as a pan-enteric tool for evaluating CD. PCCE-2 was usually performed 2 days after oral DBE. Then, we evaluated the jejunal lesions in PCCE-2 carefully, with due consideration of traumatic mucosal injury. However, suspected traumatic lesions such as reddish area and linear mucosal damage were few in the jejunum and could be differentiated with CD lesions. Whole GI tract observation was evaluated using the landmark CD lesion, fluoroscopy, and gastrografin enterography; however, the marking clip placement was considered to be more accurate for evaluation.

In conclusion, PCCE-2 is a safe and feasible tool to examine the whole GI tract in patients with CD with a high diagnostic yield for CD lesions in the entire GI. Further large-scale studies are required for an in-depth understanding of the usefulness of PCCE-2 for CD.

## Supplementary information

**Additional file 1.** **Supplementary Table 1:** Diagnostic yield of PCCE-2 for gastric lesion. **Supplementary Table 2:** Univariate logistic analysis of factors influencing incomplete PCCE-2.

## Data Availability

The datasets used and/or analyzed during the current study are available from the corresponding author on reasonable request.
