# Supplementary material for: Diagnostic yield of colon capsule endoscopy for Crohn’s disease lesions in the whole gastrointestinal tract
Source: BMC Gastroenterol. 2021 Feb 16;21:75. doi: 10.1186/s12876-021-01657-0 (PMC7888071; doi:10.1186/s12876-021-01657-0)
Supplement: Supplementary file 1 — Additional file 1. Supplementary Table 1: Diagnostic yield of PCCE-2 for gastric lesion. Supplementary Table 2: Univariate logistic analysis of factors influencing incomplete PCCE-2. [file 12876_2021_1657_MOESM1_ESM.docx]

***Title***

**Diagnostic yield of colon capsule endoscopy for Crohn’s disease lesions in the whole gastrointestinal tract**

**Authors:**

Keisaku Yamada, MD^1^, Masanao Nakamura, MD, phD^1^, Takeshi Yamamura, MD, phD^2^, Keiko Maeda, MD, phD^2^, Tsunaki Sawada, MD, phD^2^, Yasuyuki Mizutani, MD, phD^1^, Eri Ishikawa MD, phD^1^, Takuya Ishikawa, MD, phD^1^, Naomi Kakushima MD, phD^1^, Kazuhiro Furukawa, MD, phD^1^, Eizaburo Ohno, MD, phD^1^, Hiroki Kawashima, MD, phD^2^, Takashi Honda, MD, phD^1^, Masatoshi Ishigami, MD, phD^1^, Mitsuhiro Fujishiro, Professor ^1^

**Affiliations:**

^1^ Department of Gastroenterology and Hepatology, Nagoya University Graduate School of Medicine. 65 Tsurumai-cho, Showa-ku, Nagoya 466-8550, Japan

^2^ Department of Endoscopy, Nagoya University Hospital. 65 Tsurumai-cho, Showa-ku, Nagoya 466-8550, Japan

TEL：81-52-744-2172, FAX：81-52-744-2180

Supplementary Table 1: Diagnostic yield of PCCE-2 for gastric lesion

|  | Erosion | Bamboo joint-like appearance |
| --- | --- | --- |
| Sensitivity | 100% (6/6) | 66.7% (2/3) |
| Specificity | 92.9% (13/14) | 100% (17/17) |
| PPV | 85.7% (6/7) | 100% (2/2) |
| NPV | 100% (13/13) | 94.4% (17/18) |
| Accuracy | 95.0% (19/20) | 95.0% (19/20) |

PPV, positive predictive value; NPV, negative predictive value; PCCE-2, the second-generation PillCam colon capsule endoscopy

| Supplementary Table 2: Univariate logistic analysis of factors influencing incomplete PCCE-2 | | | | | |
| --- | --- | --- | --- | --- | --- |
|  | | | | | |
|  | Excretion group  (n=15) | Non-excretion group  (n=5) | OR | 95% CI | *p* value |
| Age, years  Median (range) | 41 (19-67) | 30 (20-36) | 0.89 | 0.776-1.021 | 0.097 |
| Sex, male/female | 12/3 | 3/2 | 2.667 | 0.298-23.858 | 0.38 |
| BMI, %  Median (range) | 20.7 (19-33.8) | 18.6 (15.6-26.6) | 0.849 | 0.617-1.168 | 0.314 |
| Disease location,  L1/L2/L3* | 5/0/10 | 1/0/4 | 2.000 | 0.174-22.949 | 0.578 |
| Disease duration, years  Median (range) | 6 (1-22) | 13 (1-16) | 1.030 | 0.886-1.197 | 0.701 |
| History of surgery (%) | 20 (3/15) | 60 (3/5) | 6.000 | 0.671-53.681 | 0.109 |
| CDAI,  median (range) | 117.5 (3-324) | 208 (89-213) | 1.002 | 0.99-1.013 | 0.756 |
| mSES-CD** | 4 (0-25) | 16 (4-27) | 1.155 | 1.003-1.331 | 0.046 |
| Water intake during examination, ml  Median (range) | 2500 (1900-3500) | 2000 (750-2000) | 0.993 | 0.982-1.004 | 0.237 |

***L1/L2/L3: ileal/colonic/ileocolonic, Montreal classification

**mSES-CD: the score that evaluates the inflammation of the small and large bowel, in which the presence of stenosis was excluded from SES-CD, and applied it into the 3 segments of the small bowel, and combined with the 4 segments of the large bowel.

PCCE-2, second-generation PillCam colon capsule endoscopy; OR, odds ratio; CI, confidence interval; BMI, body mass index; CDAI, Crohn’s disease activity index
